# How George Floyd and COVID-19 are highlighting structural inequities for vulnerable women, children and adolescents

**DOI:** 10.1186/s12939-021-01540-0

**Published:** 2021-08-28

**Authors:** Kadi Toure, Etienne V. Langlois, Mehr Shah, Lori McDougall, Helga Fogstad

**Affiliations:** PMNCH, World Health Organization (WHO), Geneva, Switzerland

## Background

For global health practitioners, the conviction of Derek Chauvin for killing George Floyd and the disproportionate health and economic impact that COVID-19 is having on vulnerable groups is a stark reminder of how profoundly racial and ethnic discrimination and related inequities – such as poverty, limited access to education and discrimination in the jobs market – remain a root cause of poor health outcomes [1]. George Floyd was condemned to death as surely by entrenched and institutionalized racism as by his killer. His trial played out against a backdrop of continued violence against black and brown people, including women, children and adolescents in America and across the world. In addition to its impact on violent deaths, racial and ethnic discrimination often determines who is last in line for health care. This is especially so for those who bear the weight of social and economic bias – including women, children and adolescents. Yet if we do not redress inequities faced by women, children and adolescents, the world will not reach its development goals.

Even before COVID-19, research has shown that inequalities due to race and ethnicity are a fundamental cause of poor health outcomes. For example, maternal mortality in the UK is five times higher among black women and two times higher for women from Asian ethnic backgrounds than white women [2]. A study based on evidence from 7 high income countries has found black ethnicity to be associated with a higher miscarriage risk [3]. In the US, black newborn babies are three times more likely to die than white babies when looked after by white doctors [4]. In Denmark, 13% of all children are born by non-Western immigrant women. These women and their babies are more likely to get sick and die than women coming from Western countries [5, 6]. Socio-economic vulnerabilities and suboptimal care is known to contribute to these ethnic disparities [2–6].

COVID-19 has exposed and accentuated these divides in care, particularly in low-income countries. A Lancet systematic review found increases in maternal mortality and stillbirths in LMICs during the pandemic [7]. It noted that, in all settings, impact is greatest on the most vulnerable individuals in the population. For example, in Nepal, hospital deliveries decreased, most markedly among disadvantaged groups, including women in castes perceived as lower in status [8]. Socially and economically marginalised communities are hit the hardest by the knock-on effects of COVID-19, yet they have less access to safety nets. For example, data from 59 countries show that refugees and asylum seekers have been excluded from COVID-19-related social protection [9].

Inequities in health outcomes across racial and ethnic lines, are compounded with inequities across many other sectors that impact health, such as the impact of low income on the ability to afford health care [10] Differentiated levels of education also contribute to poor health outcomes for women, children and adolescents. For example, in Malaysia, private school streams are organized by ethnicity and differentiated by quality, despite government measures to desegregate schools. Ιn Brazil, Mexico and Peru, there is evidence of persistent inequality by indigenous background in education, even after controlling for social class [11]. Yet, girl’s education is known to improve family planning and enhance maternal health and child survival [12, 13].

The pernicious relationship between racism, other forms of discrimination, inequalities and health have been laid bare in this past year. It demands a search for meaningful response, and greater accountability for action. Now more than ever, acknowledging and combatting ethnic and racial disparities must be an active part of public health policy and planning approaches for increased social justice and better health outcomes in a post-pandemic world.

## Recognizing racism as a public health priority

The PMNCH Call for Action on COVID-19 asks governments everywhere to prioritize women, children and adolescents in budgeting and planning measures related to the pandemic. This increases social protection during the current crisis, as well as contributing to post-pandemic recovery and resilience [14]. Honouring George Floyd, we support the PMNCH Call to Action in highlighting three important actions and call on partners to:

1. Invest in data and evidence – to generate high-quality data, disaggregated by race, ethnicity gender, income and locality. Use these data to inform policy decisions around health and social protection, planning and policy. More research is needed on how and why racism and social inequities are perpetuated through health systems and what can be done about it.
2. Develop and finance community-based accountability systems at scale – to capture experiences of racial discrimination – including those felt by health providers – and respond meaningfully to the findings.
3. Prevent discrimination through education and protection programmes – explicitly recognizing and addressing race-related risk and vulnerability and the explicit and implicit power asymmetries that allow their perpetuation.

Steps are now being taken to combat race-related disparities in public health. A recent bill in the US Congress calls for the creation of a federal centre to develop anti-racism health policy, and a public health approach to ending policy brutality through violence-prevention programming [15].

In public health, as in other domains, Mr Floyd’s tragic death and the disruptions caused by COVID-19 have opened a window for reckoning and action. Now opened, it must not be closed until every woman, every child and every adolescent receive the justice they deserve. We all owe it to George Floyd, and to the millions like him that have and continue to suffer from racism, to make it happen.

## Data Availability

All assertions in the editorial are supported by public data sources. Full references have been made available, including where possible, web links.
